# Contrasting Linguistic and Genetic Origins of the Asian Source Populations of Malagasy

**DOI:** 10.1038/srep26066

**Published:** 2016-05-18

**Authors:** Pradiptajati Kusuma, Nicolas Brucato, Murray P. Cox, Denis Pierron, Harilanto Razafindrazaka, Alexander Adelaar, Herawati Sudoyo, Thierry Letellier, François-Xavier Ricaut

**Affiliations:** 1 Evolutionary Medicine Group, Laboratoire d’Anthropologie Moléculaire et Imagerie de Synthèse UMR-5288, Université de Toulouse, Toulouse, France; 2 Genome Diversity and Diseases Laboratory, Eijkman Institute for Molecular Biology, Jakarta, Indonesia; 3 Statistics and Bioinformatics Group, Institute of Fundamental Sciences, Massey University, Palmerston North, New Zealand; 4 Asia Institute, University of Melbourne, Melbourne, Australia; 5 Department of Medical Biology, Faculty of Medicine, University of Indonesia, Jakarta, Indonesia

## Abstract

The Austronesian expansion, one of the last major human migrations, influenced regions as distant as tropical Asia, Remote Oceania and Madagascar, off the east coast of Africa. The identity of the Asian groups that settled Madagascar is particularly mysterious. While language connects Madagascar to the Ma’anyan of southern Borneo, haploid genetic data are more ambiguous. Here, we screened genome-wide diversity in 211 individuals from the Ma’anyan and surrounding groups in southern Borneo. Surprisingly, the Ma’anyan are characterized by a distinct, high frequency genomic component that is not found in Malagasy. This novel genetic layer occurs at low levels across Island Southeast Asia and hints at a more complex model for the Austronesian expansion in this region. In contrast, Malagasy show genomic links to a range of Island Southeast Asian groups, particularly from southern Borneo, but do not have a clear genetic connection with the Ma’anyan despite the obvious linguistic association.

The Austronesian expansion was a major human migration in Southeast Asia, triggered by the spread of agricultural populations approximately 5,000 years ago^1^,^2^,^3^. Thought to have originated in Taiwan, its influence spread through Philippines and Indonesian archipelago, ultimately impacting a wide geographical area ranging from Remote Oceania in the east, to Madagascar and the eastern coast of Africa in the west^2^,^4^,^5^. This expansion had outsized cultural and genetic impact on these territories, but the populations caught up in the dispersal were regionally different and diverse across the Indo-Pacific. This created a diverse modern range of Austronesian populations with their own cultural traits and genetic heritage, among which Madagascar is a unique case.

Despite clear evidence, based on biological^6^,^7^,^8^,^9^,^10^ and linguistic data^11^,^12^, of Malagasy’s mixed ancestry with both African and Southeast Asian groups, identifying the parental populations of Malagasy and clarifying the process of settling Madagascar around the middle of the first millennium AD^13^,^14^,^15^ has remained complex. Language studies have identified many linguistic characters that relate Malagasy to languages spoken in Borneo, notably in the Southeast Barito region. This includes much vocabulary and structural linguistic agreement shared between Malagasy and Southeast Barito languages, which form a subgroup of West Malayo-Polynesian languages in the Austronesian language family^11^,^16^,^17^,^18^,^19^,^20^,^21^. Among the communities speaking Southeast Barito languages, the Ma’anyan show linguistic characteristics that place them as the closest known Asian parental population to Malagasy^16^,^17^,^18^,^22^,^23^. Curiously, the Ma’anyan are an indigenous ethnic group representing approximately 70,000 individuals, who live in remote inland areas of central and southeastern Kalimantan (the Indonesian part of the island of Borneo). Today, the Ma’anyan are largely agricultural, cultivating dry rice on shifting fields, but also gathering forest products^24^. They do not exhibit any particular mastery of seafaring technologies or navigational knowledge^22^, raising questions about how a closely related language travelled across the vast Indian Ocean and came to be spoken in Madagascar. However, in historical times, the south Borneo coastline was split by a gulf that may have extended 200 kilometres into the interior^25^,^26^, thus potentially placing Ma’anyan communities that are firmly inland today in what was then a formerly coastal environment.

Several genetic studies have sought to detect Indonesian genetic connections in the Malagasy genome (including mitochondrial DNA, Y chromosome and autosomal markers)^6^,^7^,^8^,^9^,^10^, but no clear parental groups in Southeast Asia have yet been identified. The limited geographical coverage of Indonesian populations in these studies (including the absence of key populations such as the Ma’anyan) has often prevented precise conclusions. The possibility that the Ma’anyan are the Asian parental source of Malagasy was first explored genetically using uniparental markers (mitochondrial DNA and the Y chromosome) only in 2015^10^. This preliminary study, which covered a range of Southeast Asian groups, linked the origins of the Asian genetic components in Malagasy to modern populations located between Sulawesi (eastern Indonesia) and eastern Borneo (western Indonesia), thus confirming the general results of earlier studies^8^. However, surprisingly, the Ma’anyan shared few mtDNA or Y chromosome lineages with Malagasy. Given this apparent contradiction between linguistic evidence and genetic analyses of uniparental markers, and to overcome the potential bias of this lineage-based approach (which is more sensitive to genetic drift), a genome-wide analysis of Southeast Borneo individuals was deemed necessary to better explore the link between Madagascar and Borneo.

Here, we perform that genome-wide analysis in the Ma’anyan and other groups from southern Borneo to determine the genetic background and potential Asian sources of the Malagasy. Using Illumina HumanOmniExpress Bead Chips, we genotyped over 700,000 genomic markers in 169 Ma’anyan individuals, together with a further 42 individuals from Dayak ethnic groups across southern Borneo. The aims of this study were dual: i) to examine the genetic diversity of populations in southeastern Borneo (focusing on the Ma’anyan and other indigenous Dayak groups), and thereby determine their place in the wider genetic diversity of Island Southeast Asia; and ii) to identify whether the clear linguistic relationship between the Ma’anyan and Malagasy is also reflected in a shared genetic inheritance.

## Results

### The unique Austronesian origin of the Ma’anyan

**Figure 1:**
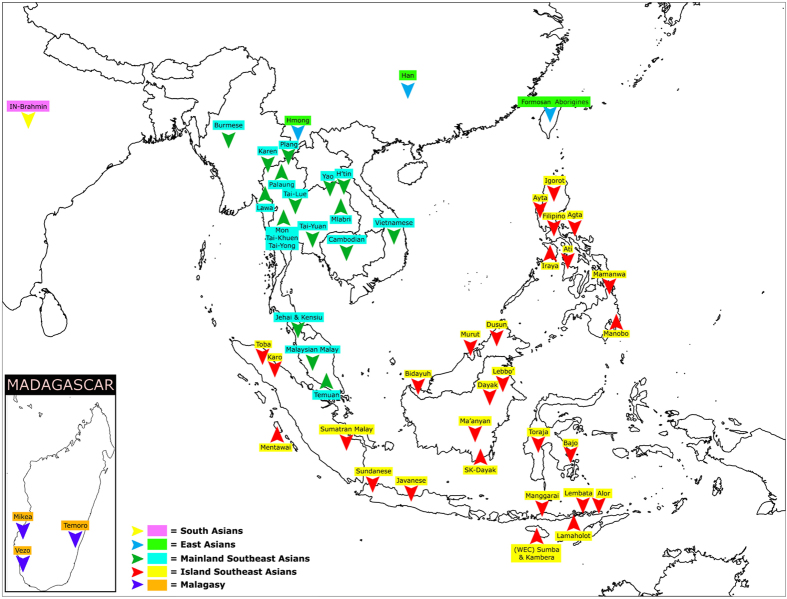
Map showing the location of each population group studied in this work. The map is generated using Global Mapper v.15 software (http://www.bluemarblegeo.com/products/global-mapper.php).

Following quality control, we obtained genotypes for 701,211 SNPs in a new set of 202 individuals from Borneo: 162 Ma’anyan and 40 South Kalimantan Dayak (SK-Dayak). To characterize the Ma’anyan and SK-Dayak gene pool within an Asian context, we focused our analyses on Island Southeast Asian, East Asian and Mainland Southeast Asian populations (Fig. 1). In a Principal Component Analysis (PCA) using a subset of the SNPs that intersect with published data from an extensive range of regional populations (the low density dataset) (Supplementary Fig. S1), the first principal component (explaining 19.3% of the variance) separates Island Southeast Asian populations from East Asian and Mainland Southeast Asian groups, while the second principal component (explaining 17.5% of the variance) splits the Igorot on the positive axis and the Ma’anyan on the negative axis, with other Austronesian-speaking populations falling in between, such as Taiwanese aborigines, Filipinos, Borneo populations (Murut, Dusun, Lebbo’ and South Kalimantan Dayak) and Sumatran populations (Sumatran Malay and Karo). Other Austronesian-speaking groups, like the Bidayuh, Javanese and Malaysians cluster towards mainland Southeast Asia, likely due to the historical influence of that region on these groups. Interestingly, the Ma’anyan form their own pole on the plot and do not cluster closely with other populations from Borneo, although the genetically closest population is still the South Kalimantan Dayak group, which is also geographically the nearest neighbour to the Ma’anyan. A similar population clustering pattern is observed with both the low- and high density SNP datasets (Supplementary Fig. S2). This observation also agrees with F_ST_ values calculated on the low density dataset (Supplementary Table S1).

**Figure 2:**
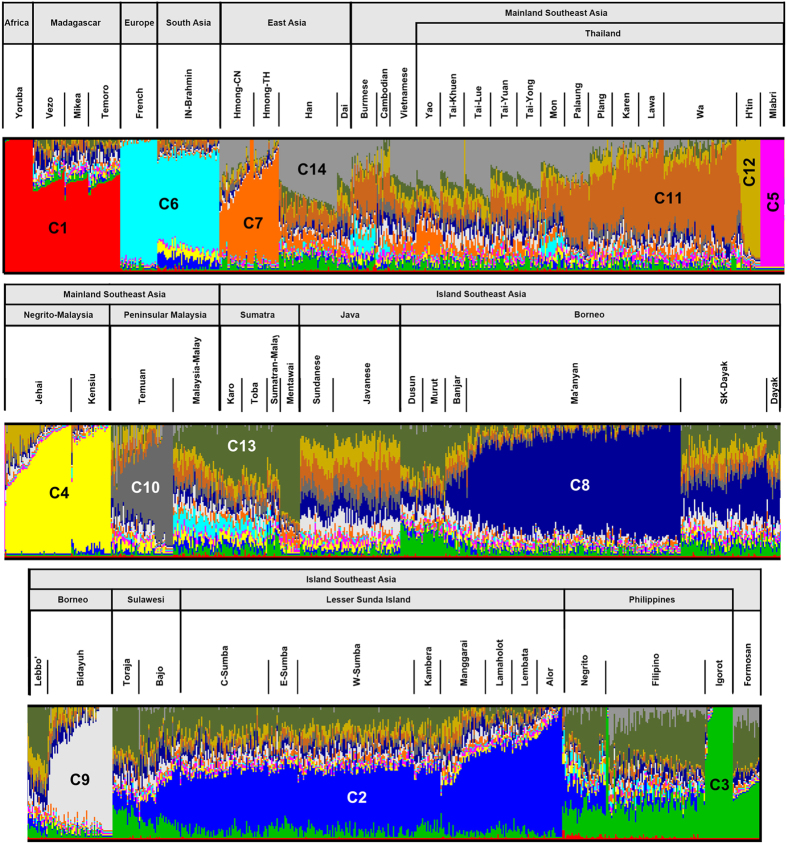
ADMIXTURE plot using the low density database with K = 14 (the optimum determined by cross-validation). Each component is identified by a specific color and a C label which corresponds to its order of appearance from K = 2 to K = 14.

This unique genetic placement of the Ma’anyan is supported by admixture estimates, also performed on the low density dataset (Fig. 2), especially at K = 14 where it achieves its lowest cross-validation value (Supplementary Fig. S3). The main ancestral components observed in Southeast Asian populations are: i) an Austronesian Igorot and indigenous Formosan component (C3; light green), ii) a Mainland Southeast Asian (MSEA) component (C11; light brown); and iii) a Papuan component (C2; light blue). However, our analysis reveals a major new component in Island Southeast Asia, representing 80% to 95% of the ancestry in Ma’anyan individuals (C8; dark blue). This Ma’anyan component is also found using an ADMIXTURE analysis on our high density SNP dataset (Supplementary Fig. S4a,b). The remaining ancestry components in the Ma’anyan also occur in most of the other Indonesian populations, and may result from shared history and/or limited gene flow between the Ma’anyan and neighbouring populations. In return, the new C8 component identified in the Ma’anyan is also found at much lower frequencies in many other Indonesian groups, reaching its highest frequency in surrounding populations of Ma’anyan in Borneo (~40%), but also appearing in some mainland Southeast Asian populations. To determine whether this distinct and homogenous genetic component in the Ma’anyan results from genetic drift (due to geographic isolation and/or endogamy), we inferred the extent of ‘Runs of Homozygosity’ (ROH) in the full high density dataset. Homozygosity in the Ma’anyan is similar to that of other Borneo populations (Supplementary Fig. S5), even though these show much higher levels of admixture (Fig. 2). However, homozygosity in the Ma’anyan is lower than in the Igorot, an isolated, indigenous Austronesian-speaking population living in the Philippine highlands. This suggests that the unusual homogeneity and unique ancestry component found in the Ma’anyan reflects the population’s migration history, rather than simply resulting from high levels of genetic drift. Genetic drift has also potentially occurred in the Igorot, and other isolated ethnic populations that exhibit low genetic diversity and have small population size (such as the Mlabri)^27^,^28^, or that show a high level of consanguinity (such as the Malay Negritos)^29^.

An f3-statistics analysis reveals more clearly that the Ma’anyan is not an admixed population (Supplementary Table S2). Defining the Ma’anyan as the daughter group, all possible combinations of populations in the low density dataset returned positive f3 statistics with Z-scores > −2, indicating no significant gene flow. In addition, a TreeMix analysis supported eight migration events, none of which involved gene flow to or from the Ma’anyan (Supplementary Fig. S6). In contrast, a migration event was supported from the basal cluster of MSEA Austroasiatic-speaking H’tin and Mlabri to the Bidayuh, a population in northwest Borneo. This suggests that MSEA gene flows reached the west of Borneo, but not the east.

**Figure 3:**
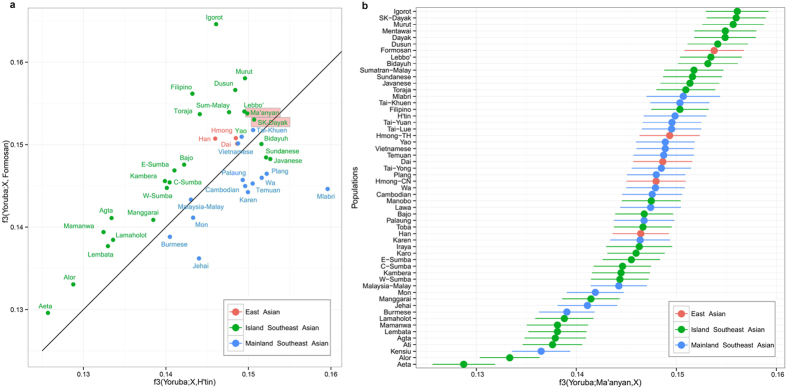
(**a**) An f3 outgroup statistics analysis showing shared genetic history with Austronesian groups (represented by indigenous Formosan) compared to Mainland Southeast Asian groups (represented by the H’tin). (**b**) Genetic similarity between Ma’anyan and other Asian populations measured using f3 outgroup statistics. Error bars show the standard error of the f3 statistics. Red dots represent East Asian groups; blue dots represent Island Southeast Asian groups; green dots represent Mainland Southeast Asian groups.

To test whether the Ma’anyan gene pool has drifted from its original Austronesian or MSEA ancestry, we performed an f3-outgroup statistics analysis (Fig. 3a). All Island Southeast Asian populations, except the Bidayuh, Javanese and Sundanese, were pulled to the Austronesian side (as defined by the Formosan aborigines). Conversely, mainland Southeast Asian groups were pulled to the MSEA side (as defined by the H’tin). The Ma’anyan fall in the upper left diagonal of the plot, indicative of genetic similarity with Austronesian rather than MSEA groups. To determine the closest population to the Ma’anyan, the configuration f3(Yoruba; Ma’anyan, *x*) was explored, where *x* represents all populations in turn in the low density dataset. The highest value was obtained when *x* was the Igorot from the Philippines or non-Ma’anyan Borneo populations (Fig. 3b), a result that is also obtained when using the high density dataset (Supplementary Table S3). These results place the genetic diversity of the Ma’anyan within the broader Austronesian gene pool.

**Figure 4:**
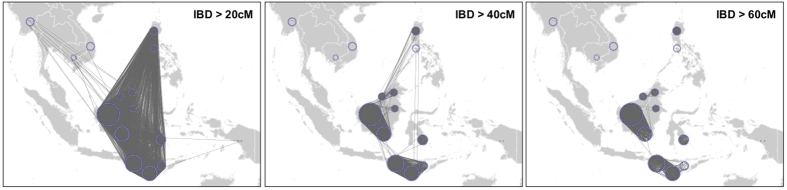
Shared Identity-By-Descent fragments between pairs of individuals in Southeast Asia, filtering for shared IBD >20 cM, 40 cM and 60 cM. Each individual is represented as a blue dot. Each individual is represented as a blue dot. Populations are represented by a circle of dots. Shared IBD fragments are represented by a black line. The maps were generated using Global Mapper v.15 software (http://www.bluemarblegeo.com/ products/global-mapper.php). The networks lines were generated using Cytoscape v.3.2.152 software (ref. 54).

This Austronesian connection is also highly supported by an Identity-by-Distance (IBD) analysis performed with Refined IBD on the high density dataset. The Ma’anyan share more haplotypes with surrounding Borneo populations and the Igorot than with Mainland Southeast Asian groups (e.g., Cambodians, Burmese and Vietnamese) (Fig. 4 and Supplementary Fig. S7). When filtered for a total shared haplotype length greater than 20 cM (~20 Mb) between two individuals, links were still observed between the Ma’anyan and Mainland Southeast Asian groups, as well as other Indonesian populations. However, the links with Mainland Southeast Asian groups disappear with larger haplotype lengths, while connections with Austronesian groups (including the Igorot) are maintained up to a threshold of 40 cM, indicating more recent common ancestry (the hypothesis of recent gene flow can be discarded from earlier analyses). At higher thresholds (i.e., longer shared haplotypes), only connections within Borneo remain. Together, these analyses (ADMIXTURE, PCA, Runs of Homozygosity, f3 statistics, TreeMix and IBD) suggest that the unique Ma’anyan genetic component is an undetected part of the broader Austronesian genetic diversity. The Ma’anyan harbour a unique Austronesian genetic component, thus allowing us to raise the question: did the Ma’anyan gene pool contribute strongly to Malagasy, as suggested by linguistic evidence?

### The Island Southeast Asian ancestries of the Malagasy

We performed PCA using the low density dataset, finding that the first two components described 54.6% of the observed variance (Supplementary Fig. S8). The first component (PC1; explaining 39.1% of the variance) largely separated the continental groups of Africa, Europe, South Asia, and East and Southeast Asia. The second component (PC2; explaining 15.5% of the variance) differentiated the Malagasy, and separated the East and Southeast Asians into a broad north-to-south gradient. The Ma’anyan and South Kalimantan Dayak populations fall within the Asian cluster. The three previously published Malagasy groups (Temoro, Vezo and Mikea) are located at an intermediate position between the African and Asian clusters, reflecting their mixture of African and Asian ancestries^9^. Overall, Malagasy appear to contain more African ancestry than Asian.

Explicit admixture analysis on the low density dataset confirms this assessment, showing that the three Malagasy populations have ~70% African ancestry (red) versus ~30% Asian ancestry (mixed colours; Fig. 2). These two main components appear consistently, in similar proportions, in plots from K = 2 to K = 14 (Supplementary Fig. S9). The Asian ancestry of Malagasy individuals is diverse, with no component (or set of components) pointing to a specific Asian population as the source of Malagasy. The Asian components found in Malagasy instead occur across Island Southeast Asia, including the South Kalimantan Dayak, Dusun, Murut, Javanese and the Ma’anyan. However, as described in the previous section, the Ma’anyan carry a particular component (C8) at very high frequency (50 to 95%), but this is much less frequent in other Western Indonesian populations (<50% in the South Kalimantan Dayak) and in the Malagasy (2–15%), which instead exhibit a balanced range of other Asian components. The PCA and ADMIXTURE analyses confirm potential connections between Malagasy and western and central Indonesian populations (particularly Java, Borneo and Sulawesi), but do not pinpoint a primary source. These results are also consistent with the general nature of Island Southeast Asian gene flow into Malagasy, as determined by TreeMix (Supplementary Fig. S10).

**Figure 5:**
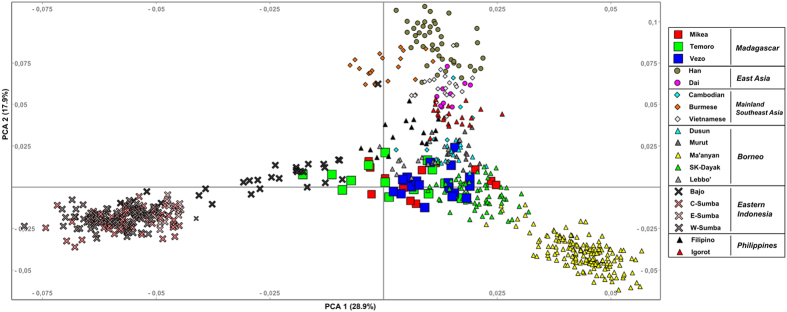
Ancestry-specific Principal Component Analysis based on masked SNPs from the high density dataset obtained after PCAdmix analysis.

Since the African ancestry in Malagasy may hinder the precise identification of Asian parental sources, we performed a PCAdmix analysis on the full high density dataset to mask African variants in the Malagasy data. An Asian ancestry-specific PCA, run on the filtered set of 17,043 SNPs, explained 46.8% of the observed variance in the dataset (Fig. 5). The first principal component separated eastern Indonesians from western Indonesians and mainland East Asians. The second principal component separated the mainland Asian groups from those in Island Southeast Asia. As observed previously (Supplementary Fig. S1), the Ma’anyan are positioned away from the other Island Southeast Asian groups and form their own pole on the graph. In this more refined analysis, the Asian markers found in the three Malagasy populations overlap closely with those from coastal Borneo (South Kalimantan Dayak, Murut and Dusun), although they do not obviously show any specific affinity with the Ma’anyan. Additionally, some Malagasy individuals are closely clustered with Bajo individuals, which may indicate that sea-nomads are relevant factors in the migrations to Madagascar, as suggested earlier^10^. Despite this general link between Malagasy Asian ancestry and Borneo groups, an F_ST_ analysis using the same dataset highlights that the South Kalimantan Dayak still have the lowest genetic distance to the three Malagasy groups (average F_ST_ = 0.022) (Supplementary Table S4), thus suggesting that this is a likely Asian source population.

Together, these analyses confirm that Malagasy are a mixture of African and Island Southeast Asian populations, as suggested by much previous research^6^,^7^,^8^,^9^,^30^,^31^. However, this study provides the new information that the Island Southeast Asian populations with closest genetic affinity to the Malagasy are located along the coasts of Borneo, although exact source populations still cannot be clearly identified. Surprisingly, the Ma’anyan, despite speaking the closest sister language to Malagasy, do not share any particularly strong genetic links with Malagasy (Figs 2 and 5). This lack of convergence between the genetic and linguistic evidence suggests that a more complex model is needed for the settlement of Madagascar. On the other hand, the uniqueness of the genetic diversity observed in the Ma’anyan opens an unexpected window for studying the complex history of the Austronesian expansion in Island Southeast Asia.

## Discussion

### A more complex picture of Austronesian genetic diversity

A genome-wide analysis of 211 individuals from Borneo reveals the unique genetic diversity of the Ma’anyan, opening an unexpected viewpoint into Southeast Asian prehistory. Our data reveal that the Ma’anyan are characterized by a specific genomic component that differentiates them from other Island Southeast Asian groups (Fig. 2 and Supplementary Fig. S1). This does not simply result from strong genetic drift (Supplementary Fig. S5), but instead represents a homogenous genetic component that is largely uninfluenced by external gene flow. Although currently living in an isolated location, the Ma’anyan only settled there recently (see details below)^24^,^32^. This recent migration to isolated inland territories appears to have favoured the preservation of a unique genetic component, which is only rarely found in other Southeast Asian populations.

Recent studies have identified at least three broad genomic classes that dominate the gene pool of Southeast Asian individuals: Papuan ancestry, Mainland Southeast Asian ancestry, and Austronesian ancestry^33^,^34^. To relate these components to major episodes of human migration inferred from previous anthropological and archaeological studies, the Papuan ancestry likely tracks back to the initial settlement period (60–45 kya)^33^,^35^,^36^, the Mainland Southeast Asian ancestry probably to the very late Pleistocene (30–10 kya)^33^,^34^,^37^,^38^, and the Austronesian ancestry to the mid-Holocene (5 kya)^33^,^34^,^35^,^36^. The discovery of a new ancestry component in the Ma’anyan is novel, although we show that it does occur at low levels in many populations across Island Southeast Asia (Fig. 2). The presence of this component in these groups does not appear to be linked to any recent admixture events (Supplementary Fig. S6 and Supplementary Table S2), and therefore might instead be the signal of ancient shared ancestry. Nevertheless, this Ma’anyan component retains links to Austronesian diversity (Fig. 3a,b), with the Ma’anyan showing a particularly close genetic connection to the Igorot in the Philippines (Figs 3b and 4). The Igorot, who also have strong Austronesian connections, live in remote areas of the Philippine highlands, which likely favoured the retention of their specific genetic signature. Shared connections between the Igorot and the Ma’anyan highlight a more complex picture of Austronesian genetic ancestry than has previously been presumed. We postulate that the ancestral diversity behind the Ma’anyan and Igorot genomic components emerged from some common unidentified source around East Asia or Taiwan, perhaps due to isolation-by-distance effects. The diffusion, and subsequent differentiation, of these two genetic components may find some support in the diffusion from Taiwan of two different cultural groups identified, respectively, by cord-marked and red-slipped pottery materials^39^,^40^. However, the modality and timing of the spread of this ancestral Ma’anyan population and its relationship to the Austronesian expansion needs to be investigated further.

### The Ma’anyan are not the primary biological ancestors of Malagasy

Despite strong linguistic affinities^11^,^16^,^17^,^20^, the Ma’anyan were not obviously the primary source population of the Malagasy. This confirms results obtained from uniparental markers, which show little sharing of genetic lineages between these two populations^10^. As hinted previously^9^, the Asian ancestry of the Malagasy is instead diverse, and appears to relate to a range of Southeast Asian populations, albeit with especially close connections to groups in southern Borneo. It seems likely that the Asian individuals who settled Madagascar were already highly mixed, rather than coming from a wide range of Asian populations with later mixing in Madagascar, in agreement with the most likely scenario whereby only a small number of migrants were involved in the initial settlement of Madagascar^41^. Looking across the Indonesian genetic landscape, the Ma’anyan carry a distinctive autosomal gene pool (dominated by the C8 component), which is not found in Malagasy (Figs 2 and 5). This marked genomic difference between the Ma’anyan and the Asian component of Malagasy contradicts the hypothesis of a common origin inferred from the languages spoken by these two groups^11^,^16^,^17^,^20^. Hence, despite the strong affinity of Ma’anyan with the Malagasy language, the Ma’anyan people apparently did not contribute significantly to the Malagasy gene pool.

Other anthropological data may shed new light on the complex history of the Ma’anyan, perhaps reconciling this discrepancy between the linguistic and genetic data. Prior to their migration to Madagascar around 1,400-1,000 years ago, proto-Malagasy people had probably already developed a derived language that differed from Ma’anyan^26^. This cultural process was likely driven by the growing influence of Malay and Javanese populations, which were trading intensively with groups in southeast Borneo^11^,^20^. The only pre-colonial record from the region, the *Hikayat Banjar* (the ‘Tale of Banjar’) describes an old Malay settlement in southern Borneo, further inland than today’s south Borneo coastline, that acted as a trading outpost of Malay Kingdom – such as the important Hindu kingdom of Srivijaya, which was dominant from the 7–13^th^ centuries AD^42^. This outpost was established because the coastline might have extended over 100 kilometres, and perhaps as much as 200 kilometres, further inland that at present^25^,^26^, and possibly laid near Tanjung-Amuntai region, the auto-identified Ma’anyan’s original homeland^24^,^32^. It is conceivable that this settlement might then have provided sea contact to what is now land-bound Ma’anyan. As the coastline move southwards, the trading post were also moved south and later formed the city of Banjarmasin, which is the dominant city, commercial state, and centre of activity in the trading network of this region. The inhabitants of Banjarmasin, the Banjar people, might then have constituted a mix of individuals from south Borneo under the cultural influence of the Malay Srivijaya kingdom. Based on this historical source, together with linguistic work on the ancestral states of the Malagasy language showing a substantial number of Malay loanwords^11^,^26^, we postulate that the Asian source population of the Malagasy constituted admixed Ma’anyan individuals (best represented in our dataset by the South Kalimantan Dayak), who lived in the Srivijaya area of influence, integrating Malay and Javanese cultural traits and favouring a large degree of gene flow, before migrating to Madagascar. Although the cause of their migration remains elusive, our data tend to favour an origin for the Malagasy in southern Borneo. Curiously, the group with the closest genetic affinity to Malagasy in our dataset is the South Kalimantan Dayak, a composite population of several ethnic groups located in southeast Borneo today (Supplementary Table S4). This suggests that an in-depth analysis of these ethnic groups, including the Banjar people and other southeast Borneo ethnic communities, might be a promising direction to better identify the (possibly mixed) genetic sources of the Malagasy and to determine the ultimate causes of the Malagasy expansion.

Our study shows that the Ma’anyan have genetic diversity that is unique in Southeast Asia, complicating existing scenarios of dispersal during the Austronesian expansion. Surprisingly, this component clearly shows that the Ma’anyan are not the primary source population of the Malagasy, as has long been supposed based on their common linguistic origin. The Asian parental population of the Malagasy instead appears to lie among the ethnic groups of the South East region of Borneo, potentially represented by the Banjar, or more generally, by the South Kalimantan Dayak people. This discrepancy between linguistic and genetic evidence may reflect the complex history of the south Borneo region, and more focused study of its peoples is needed to explore this hypothesis further.

## Methods

### Sample collection and ethics

A total of 211 DNA samples were analysed from two groups in Borneo: The Ma’anyan ethnic group (169 individuals), and the South Kalimantan Dayak, which comprises a mixed assemblage of diverse Dayak ethnic groups (42 individuals) (Fig. 1 and Supplementary Table S5). The samples used in this study have been described previously^10^. Briefly, blood samples were collected from healthy adult donors, all of whom provided written informed consent. DNA was extracted using a standard salting-out procedure. All participants were surveyed for language affiliation, current residence, familial birthplaces, and a genealogy of four generations to establish ancestry. This study was approved by the Research Ethics Commission of the Eijkman Institute for Molecular Biology (Jakarta, Indonesia), and the methods were carried out in accordance with the approved guidelines. Genome-wide SNP genotypes for the two groups were generated using the Illumina HumanOmniExpress-24 v1.0 Bead Chip (Illumina Inc., San Diego, CA), which surveys 730,525 single nucleotide markers regularly spaced across the genome. Genotyping data are available upon request.

### Dataset integration

Two datasets were compiled from previous published data to fulfil key analytical criteria: i) the low density dataset has wide geographical coverage, but includes relatively few SNPs; while ii) the high density dataset has greatly increased SNP density, but includes fewer populations. This approach, which is necessitated by the wide range of DNA genotyping chip technologies used by the scientific community (Supplementary Table S5), allows us to address the widest range of questions.

Filtering and quality controls were performed using PLINK v1.9^43^: i) to avoid close relatives, relatedness was measured between all pairs of individuals within each population using an Identity-by-Descent (IBD) estimation with upper threshold of 0.25 (second degree relatives); ii) SNPs that failed the Hardy-Weinberg exact (HWE) test (P < 10^−6^) were excluded; iii) samples with an overall call rate <0.99 and individual SNPs with missing rates >0.05 across all samples in each population were excluded; and iv) variants in high linkage disequilibrium (r^2^ > 0.5; 50 SNP sliding windows) were also removed for the low density dataset.

The final low density dataset contained 9,743 SNPs in 1,817 individuals from 73 populations, after excluding 7 Ma’anyan and 2 South Kalimantan Dayak individuals for reasons of low data quality. This low density dataset includes East and Southeast Asian populations (Mörseburg *et al.*, unpublished data), Indonesian populations including the Lebbo’ and Bajo^9^, and groups from Sumba (Cox, unpublished data), together with CEPH-HGDP data^44^, HUGO Pan-Asian SNP data^45^ and data for three Malagasy populations (Mikea, Vezo and Temoro)^9^ (Supplementary Table S5). The final high density dataset comprises a subset of the populations in the low density dataset, specifically covering 311,871 SNPs in 820 individuals from 28 populations.

### Population structure analysis

The low density dataset was analysed using the following approaches. Genetic diversity was described using pairwise F_ST_ distance calculations and Principal Components Analysis using the ‘smartpca’ algorithm of EIGENSOFT v6.0.1^46^. The Runs of Homozygosity (ROH) analysis was performed in PLINK v1.9 from the linkage-disequilibrium-pruned dataset. ADMIXTURE v1.23^47^ was used to estimate the profile of individual genomic ancestries using maximum likelihood for components K = 2 to K = 20. Ten replicates were run at each value of K with different random seeds, then merged and assessed for clustering quality using CLUMPP^48^, and the cross-validation value was calculated to determine the optimal number of genomic components (here, K = 14). ADMIXTURE and PCA plots were generated with Genesis^49^ and the results were confirmed using the high density dataset, to avoid any misinterpretation due to a potential bias driven by the density of SNPs. Gene flow between populations was investigated using two different approaches: i) SNP frequencies using TreeMix v1.12^50^, with blocks of 200 SNPs to account for linkage disequilibrium and migration edges added sequentially until the model explained 99% of the variance (the TreeMix outputs in Newick format were visualized with MEGA6^51^); and three-population (f3) statistics^52^, defining the African Yoruba population as an outgroup for the low density dataset; and ii) haplotype sharing using the Refined IBD algorithm of Beagle v.4.0^53^ visualized with Cytoscape v.3.2.1^54^ using the high density dataset to estimate the total number of shared genetic fragments (logarithm of odds ratio > 3) between each pair of individuals.

To characterize the Island Southeast Asian ancestry in Malagasy individuals, we discarded estimated African components using PCAdmix^55^. First, genome-wide SNP data from Malagasy, Yoruba and Asian samples (represented by the Ma’anyan, the Igorot and the Bajo to cover a range of Asian diversity) of the high density dataset were phased using Beagle v4.0. The Yoruba and Asian samples comprised 100 randomly selected individuals, and were defined as ‘parental’ populations compared to the Malagasy ‘daughter’ population for the purposes of the PCAdmix software. The ancestry of each defined linkage disequilibrium window was estimated by the Viterbi algorithm for each individual and used to mask all potential African SNPs. The masked Malagasy dataset was merged with the high density dataset, trimmed to 17,043 overlapping SNPs, and used to find the closest Indonesian populations that match the Malagasy Asian component using F_ST_ distances, an ancestry-specific PCA in EIGENSOFT v6.0.1 and a TreeMix analysis.

## Additional Information

**How to cite this article**: Kusuma, P. *et al.* Contrasting Linguistic and Genetic Origins of the Asian Source Populations of Malagasy. *Sci. Rep.* **6**, 26066; doi: 10.1038/srep26066 (2016).

## Supplementary Material

Supplementary Information
