# Supplementary material for: Contrasting Linguistic and Genetic Origins of the Asian Source Populations of Malagasy
Source: Sci Rep. 2016 May 18;6:26066. doi: 10.1038/srep26066 (PMC4870696; doi:10.1038/srep26066)
Supplement: Supplementary Information [file srep26066-s1.doc]

**SUPPLEMENTARY INFORMATIONS**

**TITLE**

Contrasting Linguistic and Genetic Origins of the Asian Source Populations of Malagasy

**AUTHORS**

Pradiptajati Kusuma^1,2^, Nicolas Brucato^1^, Murray P. Cox^3^, Denis Pierron^1^, Harilanto Razafindrazaka^1^, Alexander Adelaar^4^, Herawati Sudoyo^2,5^, Thierry Letellier^1^, and François-Xavier Ricaut^1^,*

**Supplementary Figure S1**. Principal Component Analysis on all East Asian/Mainland Southeast Asian/Island Southeast Asian populations from the low density dataset. Sumba populations of Eastern Indonesia are not included in this plot to omit strong Papuan polar.


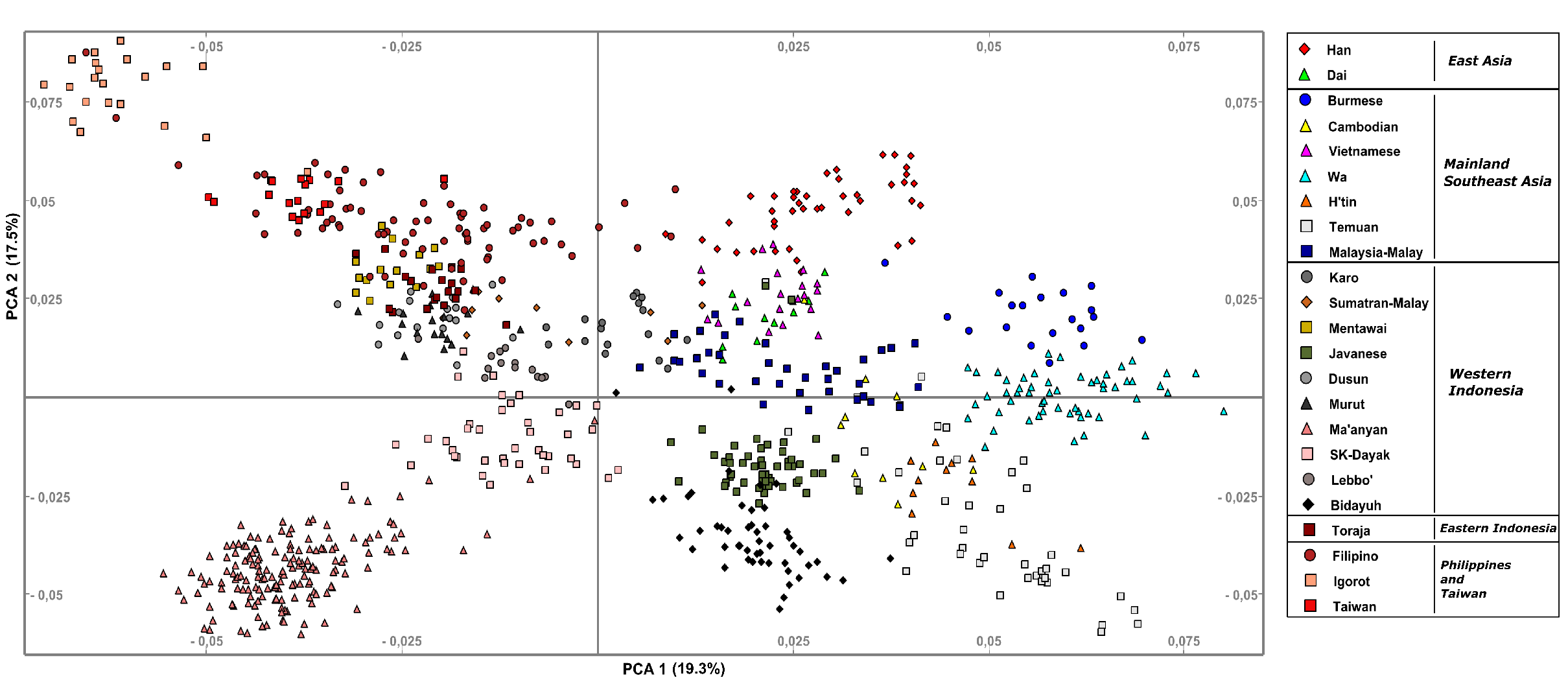


**Supplementary Figure S2.** Principal Component Analysis on East Asian/Mainland Southeast Asian/Island Southeast Asian populations from the high density dataset. Ma’anyan individuals are clustered to their own pole, clarifying unbiased population structuring in the low density dataset.


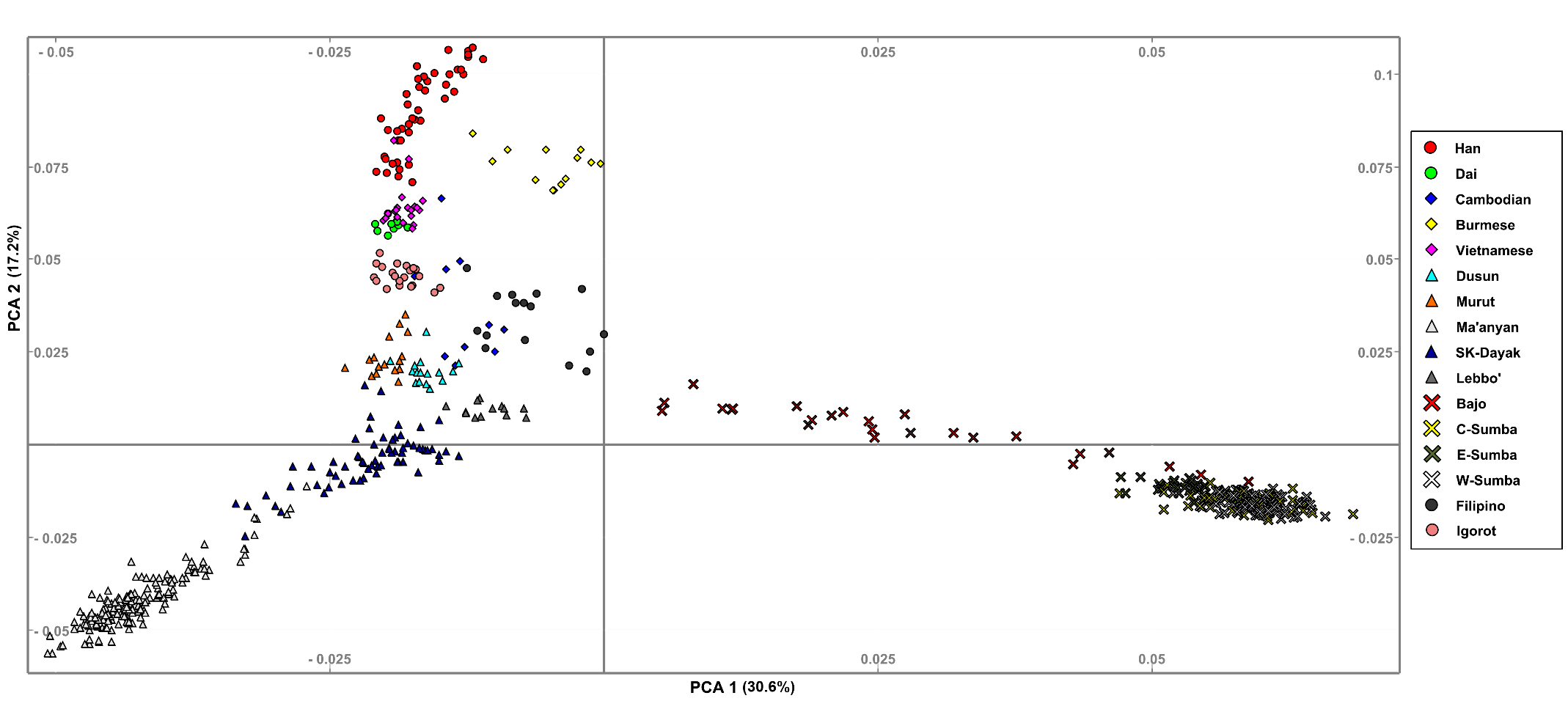


**Supplementary Figure S3**. Cross-validation plot generated from the ADMIXTURE analysis.


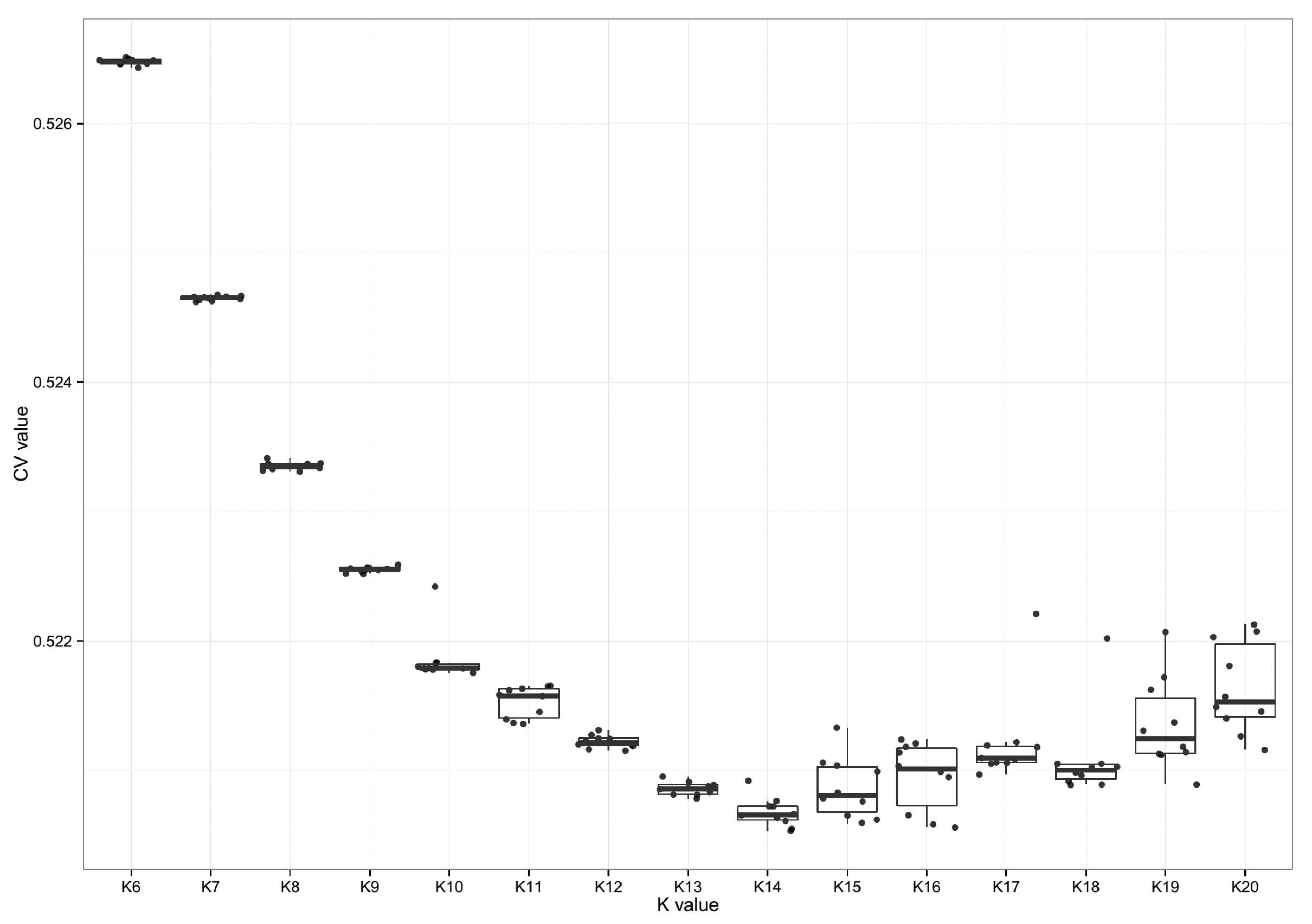


**Supplementary Figure S4.** a) ADMIXTURE plots on the high density dataset from K = 2-7 and b) its cross-validation (CV) chart showing K=6 and K=7 give the lowest CV value


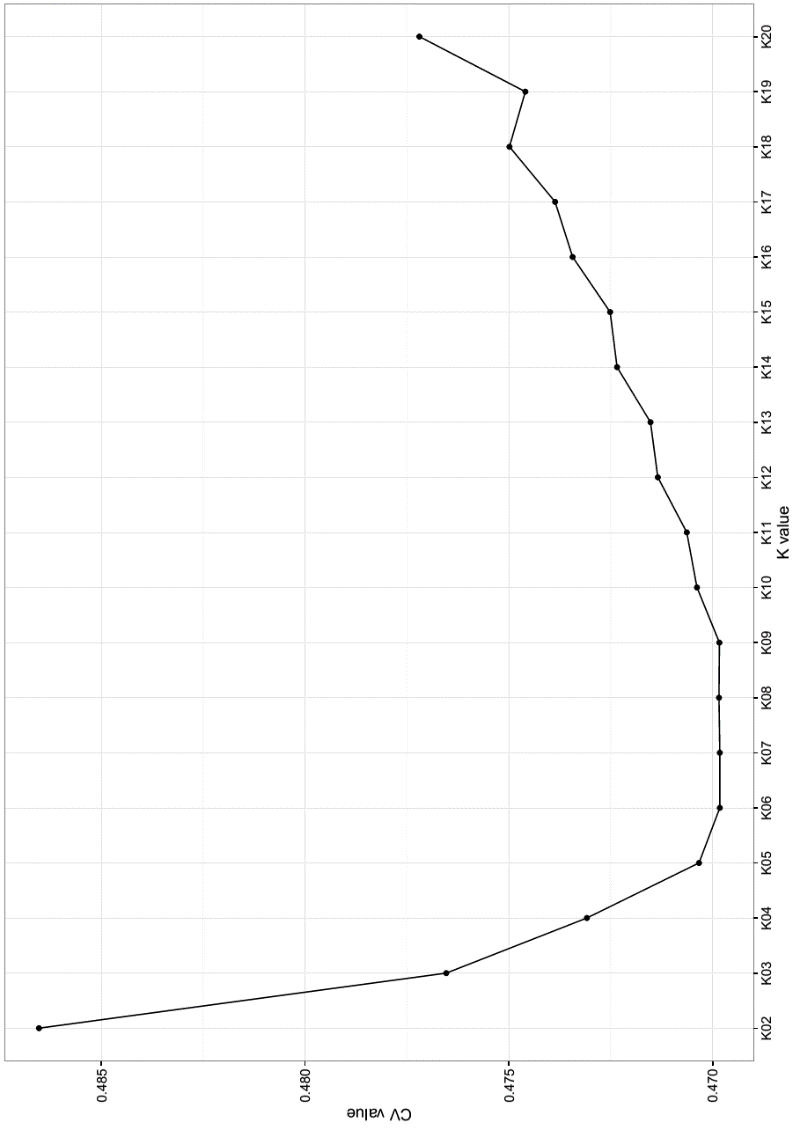

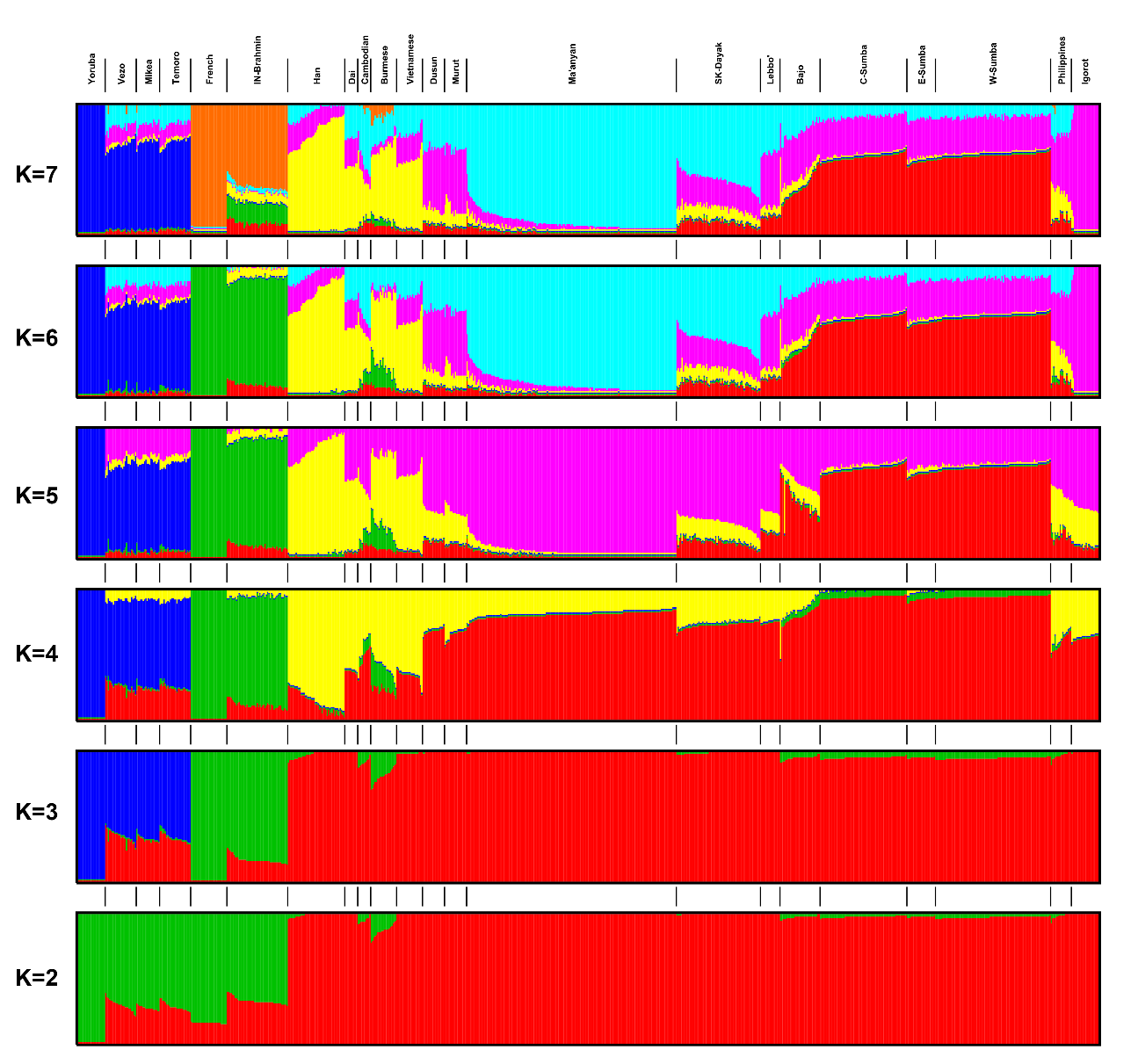


a)

b)

**Supplementary Figure S5.** Runs of Homozygosity results showing that the Ma’anyan have comparable homozygosity relative to other Borneo populations.


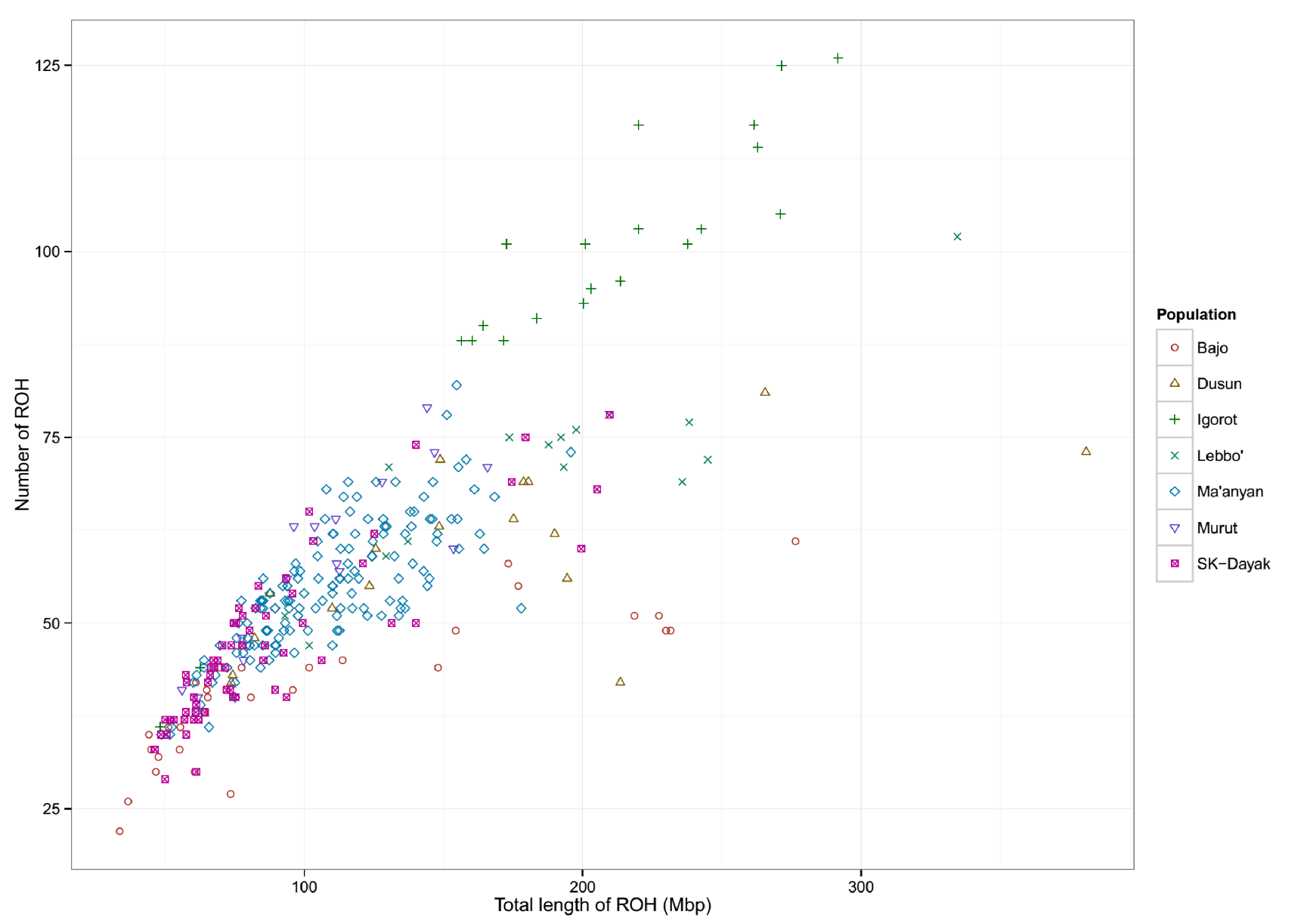


**Supplementary Figure S6**. TreeMix analysis on the low density dataset with eight migration nodes showing no gene flow to or from the Ma’anyan.


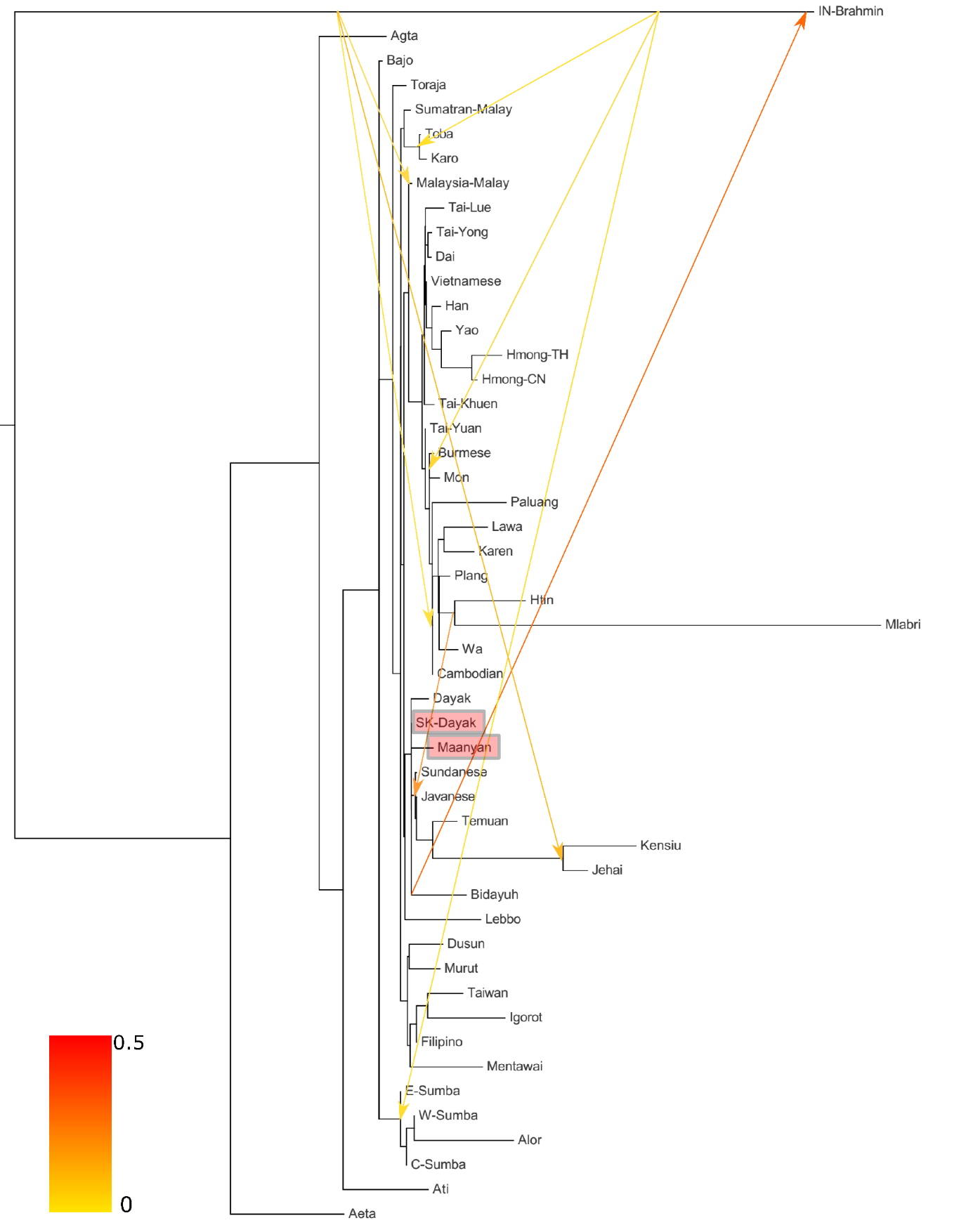


**Supplementary Figure S7**. Shared Identity-By-Descent fragments between pairs of individuals in Southeast Asia, using nine filtering thresholds (from 10cM to 90cM). The maps were generated using Global Mapper v.15 software (http://www.bluemarblegeo.com/ products/global-mapper.php). The networks lines were generated using Cytoscape v.3.2.152 software (ref. 54).


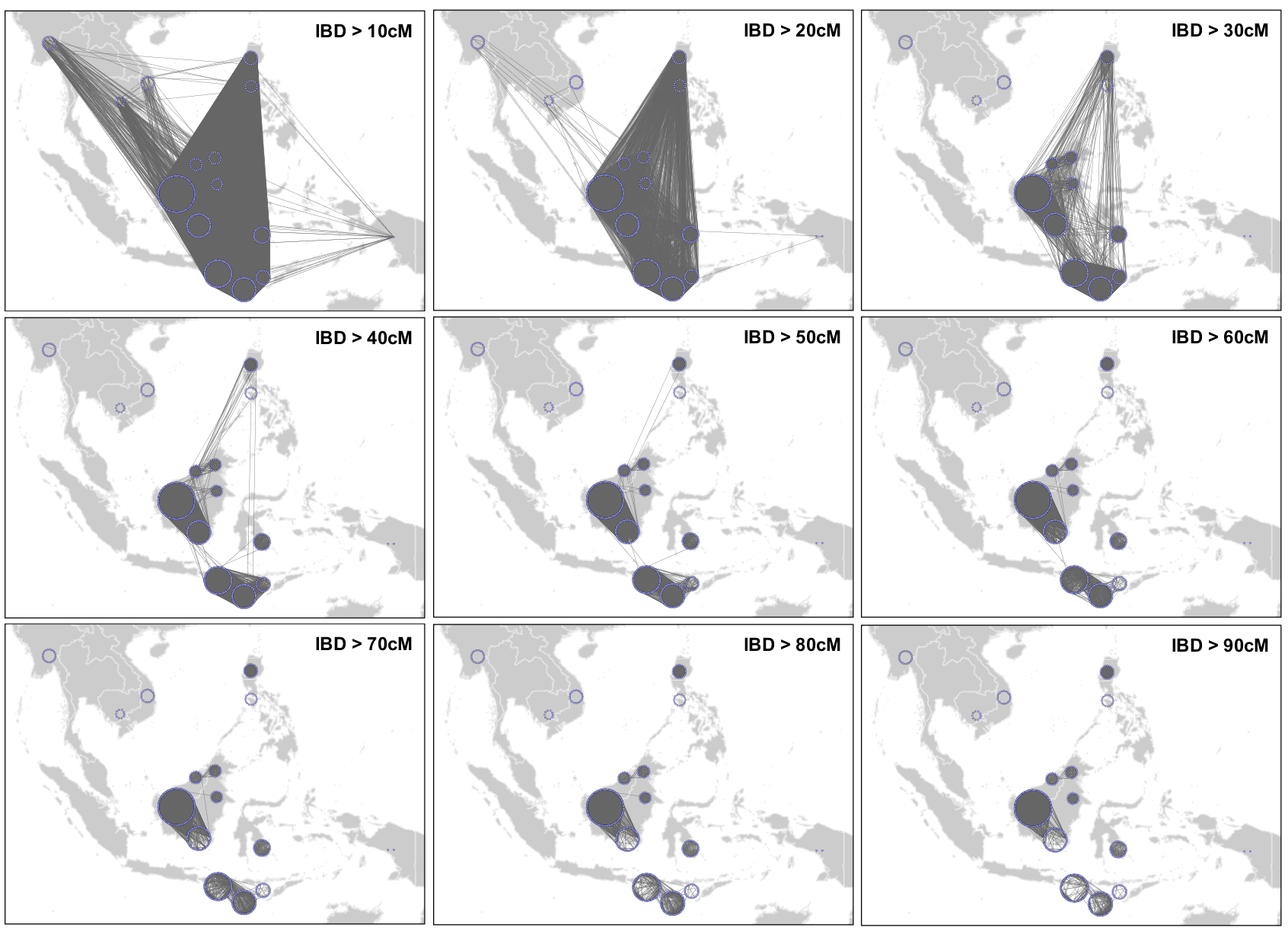


**Supplementary Figure S8**. Principal Component Analysis on all populations in the low density dataset. Ma’anyan and South Kalimantan Dayak individuals are clustered together with other Island Southeast Asian individuals.


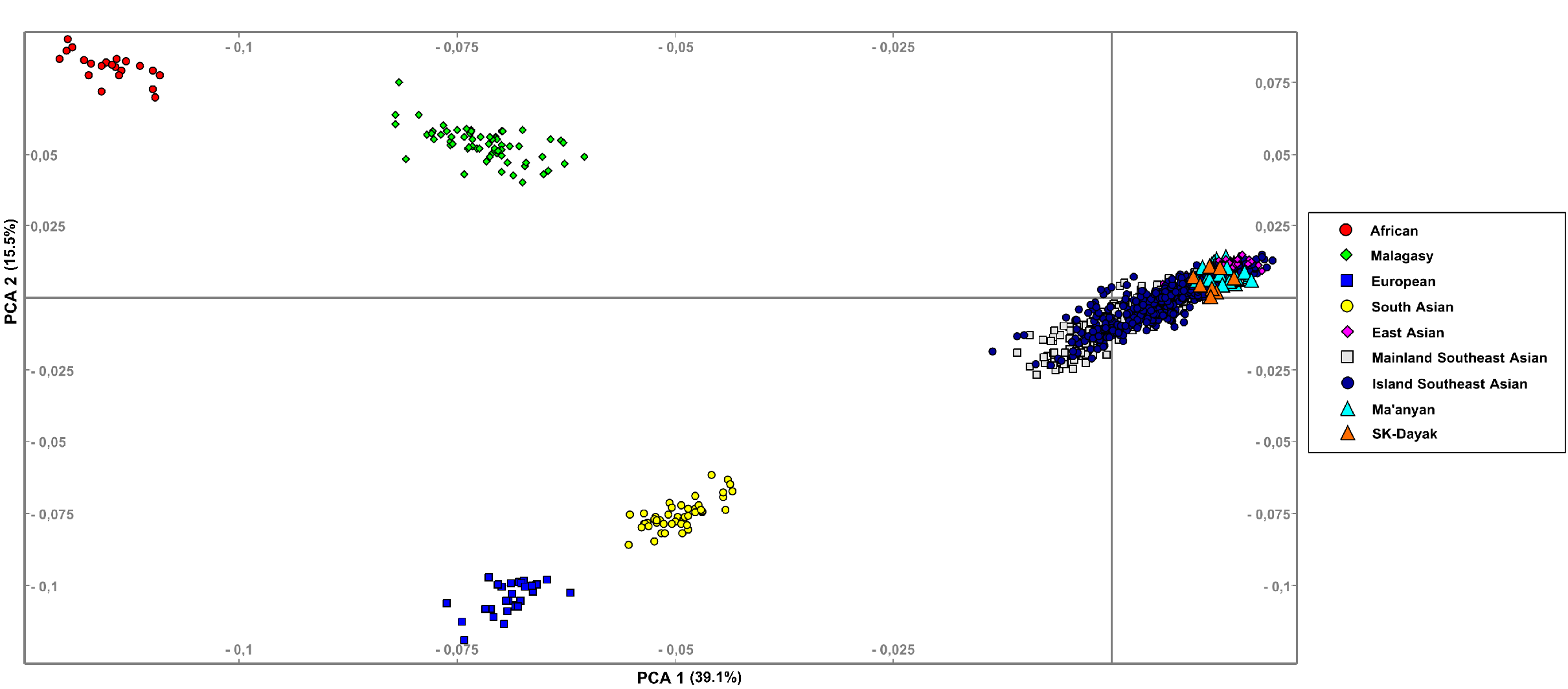


**Supplementary Figure S9**. ADMIXTURE plots on the low density dataset from K = 2-14.


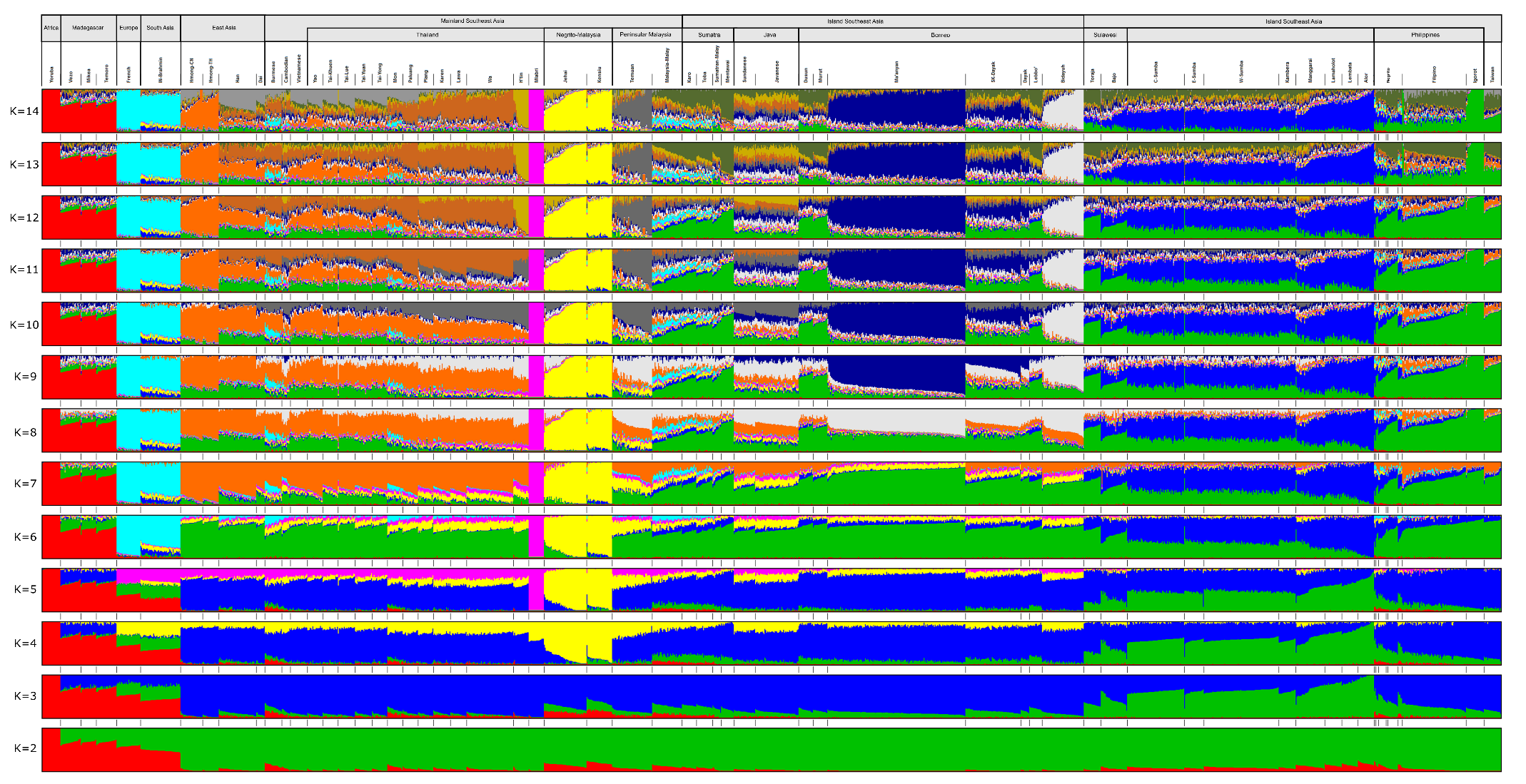


**Supplementary Figure S10**. TreeMix analysis on the low density dataset with six migration nodes showing no specific Southeast Asian gene flow into Malagasy populations.


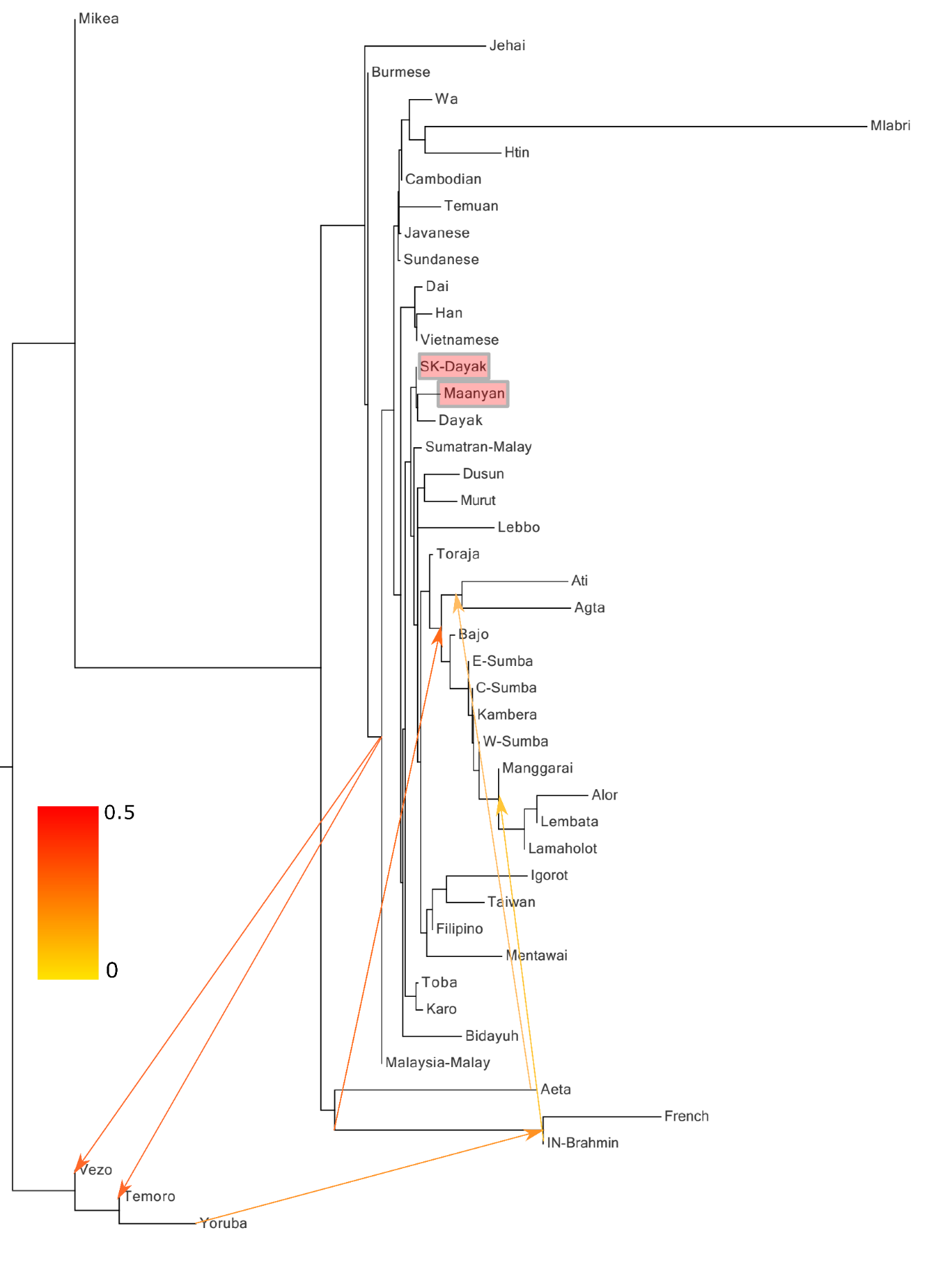


**Supplementary Table S1**. F_ST_ genetic distances between Ma’anyan and other populations in the low density dataset

| **Populations** | **Ma'anyan** |  | **Populations** | **Ma'anyan** |
| --- | --- | --- | --- | --- |
| **SK-Dayak** | 0.006 |  | **Bidayuh** | 0.026 |
| **Sundanese** | 0.012 |  | **Manobo** | 0.027 |
| **Javanese** | 0.012 |  | **Manggarai** | 0.029 |
| **Dayak** | 0.012 |  | **Taiwan** | 0.029 |
| **Cambodian** | 0.014 |  | **Karen** | 0.03 |
| **Sumatran-Malay** | 0.014 |  | **Hmong-CN** | 0.034 |
| **Malaysia-Malay** | 0.015 |  | **Lawa** | 0.034 |
| **Filipino** | 0.015 |  | **Lebbo'** | 0.034 |
| **Tai-Yuan** | 0.016 |  | **Mentawai** | 0.036 |
| **Vietnamese** | 0.017 |  | **Lamaholot** | 0.038 |
| **Toraja** | 0.017 |  | **Hmong-TH** | 0.041 |
| **Dai** | 0.018 |  | **Lembata** | 0.042 |
| **Tai-Yong** | 0.018 |  | **Igorot** | 0.042 |
| **Toba** | 0.018 |  | **Paluang** | 0.043 |
| **Tai-Khuen** | 0.019 |  | **H'tin** | 0.043 |
| **Bajo** | 0.019 |  | **Jehai** | 0.056 |
| **Karo** | 0.02 |  | **Ati** | 0.056 |
| **Murut** | 0.021 |  | **Agta** | 0.057 |
| **Burmese** | 0.022 |  | **Alor** | 0.063 |
| **Tai-Lue** | 0.022 |  | **Kensiu** | 0.073 |
| **Mon** | 0.022 |  | **IN-Brahmin** | 0.075 |
| **Plang** | 0.022 |  | **Vezo** | 0.08 |
| **Dusun** | 0.022 |  | **Temoro** | 0.081 |
| **E-Sumba** | 0.023 |  | **Mikea** | 0.085 |
| **Han** | 0.024 |  | **Iraya** | 0.087 |
| **Wa** | 0.024 |  | **Mamanwa** | 0.091 |
| **Kambera** | 0.024 |  | **Aeta** | 0.094 |
| **Yao** | 0.025 |  | **French** | 0.108 |
| **C-Sumba** | 0.025 |  | **Mlabri** | 0.154 |
| **W-Sumba** | 0.025 |  | **Yoruba** | 0.164 |
| **Temuan** | 0.026 |  |  |  |

**Supplementary Table S2**. Results of f3-statistics, showing the lowest 50 Z-score combinations, on all populations in the low density dataset to test for Ma’anyan admixture. An f3-statistic is expected to be positive significant with a Z score >-2 if no admixture has taken place.

| **Target population** | **Population 1*** | **Population 2*** | **f3 value** | **SE** | **Z-score** |
| --- | --- | --- | --- | --- | --- |
| Ma'anyan | Iraya | Mlabri | 0,0012494 | 0,000643656 | 1,9411 |
| Ma'anyan | Mlabri | Igorot | 0,000964953 | 0,000388148 | 2,48604 |
| Ma'anyan | Jehai | Igorot | 0,000531593 | 0,00021105 | 2,51879 |
| Ma'anyan | Mlabri | Taiwan | 0,000980097 | 0,000315858 | 3,10296 |
| Ma'anyan | Kensiu | Igorot | 0,00100631 | 0,000251586 | 3,99986 |
| Ma'anyan | Mlabri | Agta | 0,00216078 | 0,000539446 | 4,00555 |
| Ma'anyan | Manobo | Mlabri | 0,00146867 | 0,000360337 | 4,07582 |
| Ma'anyan | H'tin | Igorot | 0,000966092 | 0,000236618 | 4,08291 |
| Ma'anyan | Aeta | Mlabri | 0,00309591 | 0,000719123 | 4,30511 |
| Ma'anyan | Sumatran-Malay | Mlabri | 0,00241122 | 0,000508989 | 4,73727 |
| Ma'anyan | Yoruba | Igorot | 0,00206575 | 0,000392053 | 5,26905 |
| Ma'anyan | Ati | Mlabri | 0,00227472 | 0,000409701 | 5,55215 |
| Ma'anyan | Filipino | Mlabri | 0,00159951 | 0,000285164 | 5,60909 |
| Ma'anyan | Mamanwa | Mlabri | 0,00186683 | 0,000306453 | 6,09173 |
| Ma'anyan | Iraya | Jehai | 0,00209051 | 0,000343169 | 6,0918 |
| Ma'anyan | Dayak | Mlabri | 0,00207119 | 0,000331207 | 6,25346 |
| Ma'anyan | Mikea | Igorot | 0,00222687 | 0,000353957 | 6,29137 |
| Ma'anyan | Iraya | Kensiu | 0,00263222 | 0,000415146 | 6,34046 |
| Ma'anyan | Mentawai | Mlabri | 0,00214312 | 0,000337205 | 6,35555 |
| Ma'anyan | Yoruba | Mentawai | 0,00242817 | 0,000378587 | 6,41376 |
| Ma'anyan | Temuan | Igorot | 0,00127519 | 0,000198279 | 6,43128 |
| Ma'anyan | Yoruba | Murut | 0,00219002 | 0,000336151 | 6,515 |
| Ma'anyan | Mentawai | H'tin | 0,00184931 | 0,000281734 | 6,56402 |
| Ma'anyan | Iraya | H'tin | 0,00217233 | 0,000324486 | 6,69467 |
| Ma'anyan | Mlabri | E-Sumba | 0,00266682 | 0,000382994 | 6,96309 |
| Ma'anyan | Hmong-TH | Mlabri | 0,00314585 | 0,000443036 | 7,10068 |
| Ma'anyan | Dusun | Mlabri | 0,00233471 | 0,000323972 | 7,20654 |
| Ma'anyan | H'tin | Taiwan | 0,00154934 | 0,000214634 | 7,21854 |
| Ma'anyan | Vezo | Igorot | 0,00229219 | 0,000316639 | 7,23912 |
| Ma'anyan | Yoruba | Lebbo' | 0,00283852 | 0,000388072 | 7,31441 |
| Ma'anyan | Temoro | Igorot | 0,00223352 | 0,000293669 | 7,60558 |
| Ma'anyan | C-Sumba | Mlabri | 0,00269372 | 0,000352449 | 7,64286 |
| Ma'anyan | Yoruba | Mlabri | 0,0036162 | 0,000471247 | 7,67367 |
| Ma'anyan | Mlabri | Toraja | 0,00196584 | 0,000254647 | 7,71987 |
| Ma'anyan | Sumatran-Malay | Yoruba | 0,00334507 | 0,000430849 | 7,76391 |
| Ma'anyan | French | Mlabri | 0,00425063 | 0,000535803 | 7,93319 |
| Ma'anyan | H'tin | Toraja | 0,00188538 | 0,000236766 | 7,96307 |
| Ma'anyan | Mlabri | Tai-Lue | 0,00356898 | 0,000443113 | 8,05434 |
| Ma'anyan | Mlabri | Murut | 0,00249194 | 0,00030931 | 8,05645 |
| Ma'anyan | French | Igorot | 0,00228156 | 0,000282665 | 8,07162 |
| Ma'anyan | Iraya | Bidayuh | 0,00209877 | 0,000258412 | 8,1218 |
| Ma'anyan | H'tin | Agta | 0,00258133 | 0,000316796 | 8,14823 |
| Ma'anyan | Jehai | Mentawai | 0,00198762 | 0,000243679 | 8,1567 |
| Ma'anyan | Kensiu | Taiwan | 0,00151931 | 0,000184827 | 8,22014 |
| Ma'anyan | Banjar | Mlabri | 0,00253314 | 0,000308105 | 8,22168 |
| Ma'anyan | Jehai | Taiwan | 0,00131213 | 0,000159384 | 8,23251 |
| Ma'anyan | Alor | Mlabri | 0,00314537 | 0,000380185 | 8,27324 |
| Ma'anyan | Mlabri | Toba | 0,00246328 | 0,000295879 | 8,32529 |
| Ma'anyan | Hmong-TH | Yoruba | 0,00404823 | 0,000485567 | 8,33712 |
| Ma'anyan | H'tin | Dayak | 0,0021435 | 0,000257094 | 8,3374 |

* This table shows the 50 lowest Z-score combinations of populations from all combinations produced in the f3-statistics analysis

**Supplementary Table S3.** Genetic similarity between Ma’anyan and other Asian populations measured using f3 outgroup statistics on high density dataset.

| ***X*** | **f3_value** | **SE** |
| --- | --- | --- |
| Igorot | 0.213575 | 0.001511 |
| Murut | 0.211745 | 0.001481 |
| SK-Dayak | 0.211122 | 0.001447 |
| Dusun | 0.21087 | 0.001474 |
| Lebbo' | 0.209273 | 0.001471 |
| Dai | 0.208411 | 0.001502 |
| Vietnamese | 0.2072 | 0.001486 |
| Philippines | 0.206612 | 0.001473 |
| Han | 0.205027 | 0.001478 |
| Cambodian | 0.202133 | 0.001471 |
| Bajo | 0.201017 | 0.001433 |
| E-Sumba | 0.198898 | 0.001425 |
| C-Sumba | 0.198052 | 0.001435 |
| W-Sumba | 0.19762 | 0.001428 |
| Burmese | 0.192747 | 0.00141 |

**Supplementary Table S4**. Pairwise FST values from the dataset of Asian SNPs

|  | **Mikea** | **Temoro** | **Vezo** | **Malagasy (average)** |
| --- | --- | --- | --- | --- |
| Mikea | 0 | 0,018 | 0,014 |  |
| Temoro | 0,018 | 0 | 0,013 |  |
| Vezo | 0,014 | 0,013 | 0 |  |
| Han | 0,04 | 0,029 | 0,033 | 0,034 |
| Dai | 0,035 | 0,025 | 0,027 | 0,029 |
| Cambodian | 0,033 | 0,024 | 0,025 | 0,027 |
| Burmese | 0,035 | 0,025 | 0,029 | 0,030 |
| Vietnamese | 0,035 | 0,024 | 0,026 | 0,028 |
| Dusun | 0,04 | 0,031 | 0,031 | 0,034 |
| Murut | 0,039 | 0,029 | 0,03 | 0,033 |
| Ma'anyan | 0,032 | 0,024 | 0,025 | 0,027 |
| SK-Dayak | 0,027 | 0,018 | 0,02 | 0,022 |
| Lebbo' | 0,048 | 0,04 | 0,043 | 0,044 |
| Bajo | 0,033 | 0,023 | 0,026 | 0,027 |
| C-Sumba | 0,035 | 0,026 | 0,028 | 0,030 |
| E-Sumba | 0,041 | 0,029 | 0,033 | 0,034 |
| W-Sumba | 0,038 | 0,027 | 0,031 | 0,032 |
| Filipino | 0,03 | 0,02 | 0,023 | 0,024 |
| Igorot | 0,057 | 0,051 | 0,051 | 0,053 |

**Supplementary Table S5**. Populations used in the study.

| **Population** | **No. of Individuals** | **Grouped as** | **Location** | **Region** | **References** | **LDD*** | **HDD*** |
| --- | --- | --- | --- | --- | --- | --- | --- |
| Yoruba | 21 | Yoruba | Nigeria | Africa | Ref. 44 | v | v |
| French | 28 | French | France | Europe | Ref. 44 | v | v |
| Cambodian | 10 | Cambodian | Cambodia | East Asia | Ref. 44 | v | v |
| Han | 44 | Han | China | East Asia | Ref. 44 | v | v |
| Dai | 10 | Dai | China | East Asia | Ref. 44 | v | v |
|  |  |  |  |  |  |  |  |
| Vezo | 24 | Vezo | Madagascar | Africa | Ref. 9 | v | v |
| Mikea | 18 | Mikea | Madagascar | Africa | Ref. 9 | v | v |
| Temoro | 24 | Temoro | Madagascar | Africa | Ref. 9 | v | v |
| Lebbo | 15 | Lebbo | Borneo | Island Southeast Asia | Ref. 9 | v | v |
| Bajo | 31 | Bajo | Sulawesi | Island Southeast Asia | Ref. 9 | v | v |
|  |  |  |  |  |  |  |  |
| Brahmin | 47 | Brahmin | India | South Asia | Mörseburg et al., unpublished data | v | v |
| Vietnamese | 20 | Vietnamese | Singapore | Mainland Southeast Asia | Mörseburg et al., unpublished data | v | v |
| Burmese | 20 | Burmese | Singapore | Mainland Southeast Asia | Mörseburg e tal., unpublished data | v | v |
| Dusun | 17 | Dusun | Borneo | Island Southeast Asia | Mörseburg et al., unpublished data | v | v |
| Murut | 17 | Murut | Brunei | Island Southeast Asia | Mörseburg et al., unpublished data | v | v |
| Luzon | 12 | Filipino | Philippines | Island Southeast Asia | Mörseburg et al., unpublished data | v | v |
| Visayas | 4 | Filipino | Philippines | Island Southeast Asia | Mörseburg et al., unpublished data | v | v |
| Igorot | 21 | Igorot | Philippines | Island Southeast Asia | Mörseburg et al., unpublished data | v | v |
|  |  |  |  |  |  |  |  |
| Ami | 10 | Taiwan-Aborigine | Taiwan | East Asia | Ref. 45 | v | - |
| Atayal | 10 | Taiwan-Aborigine | Taiwan | East Asia | Ref. 45 | v | - |
| Wa | 56 | Wa | China | East Asia | Ref. 45 | v | - |
| Hmong | 26 | Hmong-CN | China | East Asia | Ref. 45 | v | - |
| Hmong | 20 | Hmong-TH | Thailand | Mainland Southeast Asia | Ref. 45 | v | - |
| Karen | 20 | Karen | Thailand | Mainland Southeast Asia | Ref. 45 | v | - |
| Lawa | 19 | Lawa | Thailand | Mainland Southeast Asia | Ref. 45 | v | - |
| Mlabri | 18 | Mlabri | Thailand | Mainland Southeast Asia | Ref. 45 | v | - |
| Mon | 19 | Mon | Thailand | Mainland Southeast Asia | Ref. 45 | v | - |
| Paluang | 18 | Paluang | Thailand | Mainland Southeast Asia | Ref. 45 | v | - |
| Plang | 18 | Plang | Thailand | Mainland Southeast Asia | Ref. 45 | v | - |
| TaiKhuen | 19 | TaiKhuen | Thailand | Mainland Southeast Asia | Ref. 45 | v | - |
| TaiLue | 20 | TaiLue | Thailand | Mainland Southeast Asia | Ref. 45 | v | - |
| H'tin | 18 | H'tin | Thailand | Mainland Southeast Asia | Ref. 45 | v | - |
| TaiYuan | 20 | TaiYuan | Thailand | Mainland Southeast Asia | Ref. 45 | v | - |
| TaiYong | 18 | TaiYong | Thailand | Mainland Southeast Asia | Ref. 45 | v | - |
| Yao | 19 | Yao | Thailand | Mainland Southeast Asia | Ref. 45 | v | - |
| Jehai | 50 | Jehai | Malaysia | Mainland Southeast Asia | Ref. 45 | v | - |
| Kensiu | 30 | Kensiu | Malaysia | Mainland Southeast Asia | Ref. 45 | v | - |
| Malaysian-Malay | 38 | Malaysian-Malay | Malaysia | Mainland Southeast Asia | Ref. 45 | v | - |
| Temuan | 49 | Temuan | Malaysia | Mainland Southeast Asia | Ref. 45 | v | - |
| Bidayuh | 50 | Bidayuh | Malaysia | Island Southeast Asia | Ref. 45 | v | - |
| Alorese | 19 | Alorese | Indonesia | Island Southeast Asia | Ref. 45 | v | - |
| Dayak | 12 | Dayak | Indonesia | Island Southeast Asia | Ref. 45 | v | - |
| Javanese-Jakarta | 34 | Javanese | Indonesia | Island Southeast Asia | Ref. 45 | v | - |
| Javanese-Dieng | 19 | Javanese | Indonesia | Island Southeast Asia | Ref. 45 | v | - |
| BatakKaro | 17 | Karo | Indonesia | Island Southeast Asia | Ref. 45 | v | - |
| Lamaholot | 20 | Lamaholot | Indonesia | Island Southeast Asia | Ref. 45 | v | - |
| Lembata | 19 | Lembata | Indonesia | Island Southeast Asia | Ref. 45 | v | - |
| Sumatran-Malay | 12 | Sumatran-Malay | Indonesia | Island Southeast Asia | Ref. 45 | v | - |
| Mentawai | 15 | Mentawai | Indonesia | Island Southeast Asia | Ref. 45 | v | - |
| Manggarai | 36 | Manggarai | Indonesia | Island Southeast Asia | Ref. 45 | v | - |
| Kambera | 20 | Kambera | Indonesia | Island Southeast Asia | Ref. 45 | v | - |
| Sunda | 25 | Sunda | Indonesia | Island Southeast Asia | Ref. 45 | v | - |
| BatakToba | 20 | Toba | Indonesia | Island Southeast Asia | Ref. 45 | v | - |
| Toraja | 20 | Toraja | Indonesia | Island Southeast Asia | Ref. 45 | v | - |
| Ayta | 8 | Ayta | Philippines | Island Southeast Asia | Ref. 45 | v | - |
| Agta | 8 | Agta | Philippines | Island Southeast Asia | Ref. 45 | v | - |
| Ati | 23 | Ati | Philippines | Island Southeast Asia | Ref. 45 | v | - |
| Iraya | 9 | Iraya | Philippines | Island Southeast Asia | Ref. 45 | v | - |
| Manobo | 18 | Manobo | Philippines | Island Southeast Asia | Ref. 45 | v | - |
| Mamanwa | 19 | Mamanwa | Philippines | Island Southeast Asia | Ref. 45 | v | - |
| Ilocano | 20 | Filipino | Philippines | Island Southeast Asia | Ref. 45 | v | - |
| Visaya,Chabakano | 20 | Filipino | Philippines | Island Southeast Asia | Ref. 45 | v | - |
| Tagalog | 19 | Filipino | Philippines | Island Southeast Asia | Ref. 45 | v | - |
|  |  |  |  |  |  |  |  |
| Kodi | 30 | West Sumba | Indonesia | Island Southeast Asia | Cox, unpublished data | v | v |
| Lamboya | 30 | West Sumba | Indonesia | Island Southeast Asia | Cox, unpublished data | v | v |
| Loli | 30 | West Sumba | Indonesia | Island Southeast Asia | Cox, unpublished data | v | v |
| Wanokaka | 31 | West Sumba | Indonesia | Island Southeast Asia | Cox, unpublished data | v | v |
| Anakalang | 30 | Central Sumba | Indonesia | Island Southeast Asia | Cox, unpublished data | v | v |
| Wunga | 30 | Central Sumba | Indonesia | Island Southeast Asia | Cox, unpublished data | v | v |
| Mamboro | 30 | Central Sumba | Indonesia | Island Southeast Asia | Cox, unpublished data | v | v |
| Rindi | 24 | East Sumba | Indonesia | Island Southeast Asia | Cox, unpublished data | v | v |
|  |  |  |  |  |  | v | v |
| Ma'anyan | 162 | Ma'anyan | Borneo | Island Southeast Asia | this study | v | v |
| South Kalimantan Dayak | 40 | SK-Dayak | Borneo | Island Southeast Asia | this study | v | v |

Note: * Data from this publication is used only on low density dataset (9,742 SNPs), not on high density dataset (311,871 SNPs).
